# Molecular Dynamics and Structure of Poly(Methyl Methacrylate) Chains Grafted from Barium Titanate Nanoparticles

**DOI:** 10.3390/molecules27196372

**Published:** 2022-09-27

**Authors:** Aleksandra Wypych-Puszkarz, Onur Cetinkaya, Jiajun Yan, Ruslana Udovytska, Jarosław Jung, Jacek Jenczyk, Grzegorz Nowaczyk, Stefan Jurga, Jacek Ulański, Krzysztof Matyjaszewski, Joanna Pietrasik, Marcin Kozanecki

**Affiliations:** 1 Department of Molecular Physics, Lodz University of Technology, Żeromskiego 116, 90-924 Lodz, Poland; 2 Department of Pharmaceutical Chemistry, Faculty of Pharmacy, Kocaeli Health and Technology University, 41275 Kocaeli, Turkey; 3 Department of Chemistry, Carnegie Mellon University, 4400 Fifth Avenue, Pittsburgh, PA 15213, USA; 4 NanoBioMedical Centre, Adam Mickiewicz University, Wszechnicy Piastowskiej 3, 61-614 Poznan, Poland; 5 Institute of Polymer and Dye Technology, Lodz University of Technology, Stefanowskiego 16, 90-537 Lodz, Poland

**Keywords:** nanocomposites, polymer brushes, molecular dynamics, dielectric properties

## Abstract

Core−shell nanocomposites comprising barium titanate, BaTiO_3_ (BTO), and poly(methyl methacrylate) (PMMA) chains grafted from its surface with varied grafting densities were prepared. BTO nanocrystals are high-k inorganic materials, and the obtained nanocomposites exhibit enhanced dielectric permittivity, as compared to neat PMMA, and a relatively low level of loss tangent in a wide range of frequencies. The impact of the molecular dynamics, structure, and interactions of the BTO surface on the polymer chains was investigated. The nanocomposites were characterized by broadband dielectric and vibrational spectroscopies (IR and Raman), transmission electron microscopy, differential scanning calorimetry, and nuclear magnetic resonance. The presence of ceramic nanoparticles in core–shell composites slowed down the segmental dynamic of PMMA chains, increased glass transition temperature, and concurrently increased the thermal stability of the organic part. It was also evidenced that, in addition to segmental dynamics, local β relaxation was affected. The grafting density influenced the self-organization and interactions within the PMMA phase, affecting the organization on a smaller size scale of polymeric chains. This was explained by the interaction of the exposed surface of nanoparticles with polymer chains.

## 1. Introduction

Modern electronic devices demand dielectric materials with precisely controlled properties, such as those needed for gate dielectrics, embedded capacitors, or electrostrictive actuators. Nanostructured hybrid dielectrics, i.e., organic–inorganic systems, have attracted a lot of attention and have been extensively developed. Synergistically, they combine high permittivity and high breakdown strength, characteristic of inorganic phases, with mechanical flexibility and processability typical for organic polymers. Because both components are insulators in nature, the obtained products exhibit low dielectric loss. However, ceramics with high dielectric constants cannot be mixed with a polymer matrix by simple melt blending or solution mixing because of their poor miscibility. To overcome this limitation, the organic and inorganic components should be covalently bonded at a molecular level for better control of the microstructure of obtained products. Both factors are crucial for dielectric loss in the final product [1,2,3,4,5,6].

A few strategies have been employed to prepare hybrid dielectrics with enhanced values of dielectric permittivity and low dielectric loss. Modification of the surface of the particles and the grafting density of the polymer chains by reversible deactivation radical polymerizations initiated from the surface of inorganic particles have been very efficient in controlling the microstructure of nanocomposites [7,8,9,10,11,12]. Reversible deactivation radical polymerizations, including atom transfer radical polymerization (ATRP), were used efficiently to synthesize well-defined core–shell systems with a polymer layer surrounding inorganic particles [13,14,15,16]. Such core–shell systems are termed polymer brushes. An approach in which polymer chains grow from the surface is termed the ‘grafting-from’ strategy and leads to structures in which each polymer chain is attached to the surface of the inorganic particle by one chain end. The ‘grafting from’ strategy allows the precise control of the number of grafted polymer chains and the achievement of a high grafting density (especially on curved surfaces such as the nanoparticle surface). This is because the polymerization starts from the initiators immobilized earlier on the modified surface. If the polymerization proceeds with good control, the concurrent chain growth is observed, leading to a uniform polymer layer with a predetermined molecular weight and a narrow molecular weight distribution. Alternatively, the ‘grafting-onto’ approach could be applied to produce hybrid organic–inorganic nanoparticles [17,18]. In this case, the already pre-synthesized polymers are immobilized directly on the particle surface through the functional groups located at the polymer chain end. Because the steric hindrance increases with the increasing number of polymer chains attached to the particle’s surface, as well as with the polymer molecular weight, the grafting density reached using the ‘grafting-onto’ strategy is significantly smaller when compared to the ‘grafting-from’ approach [14].

The molecular dynamics of polymer chains grafted onto stiff inorganic surfaces can significantly differ from that exhibited by free-standing macromolecules in melt or in solution. First, one of the ends of the chain is anchored on the surface, which reduces the number of degrees of freedom of the entire macromolecule. Next, the profile density of the polymer segments in the core zone differs from that characteristic of the statistical coil, which is a preferred conformation for nonionic polymer chains in melt or in solution. Additional parameters can influence the molecular dynamics of grafted polymer chains. One of the most important factors is the grafting density, which determines the growth of the polymer chain and, finally, the degree of its straightening [19]. For dense systems, the polymer chains can grow only parallelly; thus, segmental mobility is significantly reduced due to steric hindrance [20]. Furthermore, interactions within the core–core and core–shell zones are strongly influenced by the grafting density [13]. Another crucial factor is the curvature of the surface, which plays an important role because it impacts the mass distribution and density profile of the polymer segments [21,22,23,24,25,26,27].

One of the most useful techniques for studying the molecular dynamics of polymers is broadband dielectric spectroscopy (BDS). It allows for analyzing various relaxation processes and their thermal and frequency dependences. A broad range of accessible frequencies, from 0.01 Hz up to GHz, and temperatures up to 500 K, cover the fast local relaxations of small side groups through the segmental motion up to the cooperative movements of the bigger part of the macromolecule or polymer network. Each relaxation process has its characteristic relaxation time τ at a given temperature. Analysis of temperature-dependent relaxation times allows for the determination of the activation energies of relaxation processes. Usually, the slowest process detected by BDS corresponds to the motion of the whole macromolecule, and it is called the normal mode. This can be detected for polymers having a dipole moment parallel to the chain backbone (type-A chains). Then, a faster relaxation process appears due to cooperative movements of polymer chains related to the glass transition phenomena. Other relaxations occur at lower temperatures, and they relate to the local movements of specific molecular groups. The results of BDS are often supported and completed by the results of other dynamic techniques, such as dynamic mechanical analysis, temperature-modulated differential scanning calorimetry, nuclear magnetic resonance spectroscopy, and computer simulations. 

In this work, the molecular dynamics of hybrid dielectrics consisting of poly(methyl methacrylate) (PMMA) grafted from the surface of barium titanate–BaTiO_3_ (BTO) nanoparticles (NPs) were studied using BDS and solid-state ^1^H NMR spectroscopy. NMR spectra were also used to define the tacticity of PMMA grafted from BTO, while high-resolution transmission electron microscopy (HR-TEM) measurements were performed to investigate the shape and surface of the BTO-*g*-PMMA composites. Additionally, differential scanning calorimetry (DSC) and thermogravimetry (TGA) were used to assess the thermal properties of the obtained hybrid nanomaterials, including their thermal stability, while TG was also used to determine the inorganic phase content. Furthermore, vibrational spectroscopy was applied to determine interactions between organic–organic and organic–inorganic components that can influence the molecular dynamics and microstructure of core–shell nanocomposites. 

All results were analyzed and discussed considering the varied grafting density of polymer chains on spherical ceramic surfaces resulting from the different content of inorganic phase in the composite, the organization of polymer chains, and tacticity as well as interactions in core–shell systems. The increase in the grafting density is connected with a denser packing of the tethered chains on the ceramic surface, which results in their different spatial organization, i.e., transformation into brush-like conformation. Elevated values of grafting densities may cause a straightening of the macromolecules and an increase in the dipolar interactions between the polymeric chains. This, in turn, results in changes in the molecular dynamics of the grafted polymers and affects their physicochemical properties. The hypothesis behind our research was that the so-called “free” unmodified inorganic surface of ceramic is an important component affecting the molecular mobility and properties of the organic phase in organic–inorganic composites.

Recognition of molecular motions in the hybrid nanocomposites, as well as the factors that influence them, provides insight into the structure–dielectric property relationship, which is important for the design and prediction of properties of new dielectric materials for electronics.

## 2. Materials and Methods

### 2.1. Materials

Barium titanate particles (200 nm, tetragonal) were purchased from US Nano. Methyl methacrylate (MMA, Aldrich, 99%) was purified by elution through a basic alumina column to remove inhibitors. 2,2′-Azobisisobutyronitrile (AIBN, Aldrich, 98%) was recrystallized from ethanol following literature procedures [28]. Tris[2 -(dimethylamino)ethyl]amine, (Me_6_TREN, Alfa, 99%), copper (II) bromide (CuBr_2_, Aldrich, 99%), anisole (Aldrich, 99%), methanol (EMD, 99.8%) and dimethylformamide (Fisher, 99.8%) were used as received. 12-(2-bromo isobutyramido)dodecanoic acid, BiBADA was synthesized according to literature procedures [29].

Linear PMMA polymer, 350,000 g/mol, was used as a reference sample (weight-average molecular weight *M_w_* = 350,000, CAS No. 9011-14-7, Cat. No. 44,574-6 Aldrich Chemical Co.).

#### 2.1.1. Synthesis of Hybrid Nanoparticles (Grafting of PMMA from a Surface of BTO Nps)

BTO particles were modified using BiBADA as an initiator. Initiators for continuous activator regeneration (ICAR) or supplemental activator and reducing agent atom transfer radical polymerization (SARA ATRP) were used as polymerization methods to generate polymer brushes with varied grafting densities and the number average molecular weight of tethered PMMA chains. The high (1.25 chains/nm^2^) and low (0.43 chains/nm^2^) grafting density was investigated in a case of PMMA with molecular weight *M*_n_ < 200,000 (Table 1). Furthermore, for PMMA brushes with low grafting densities, *M*_n_ was varied from 185,000 (0.43 chains/nm^2^) to 318,000 g/mol (0.18 chains/nm^2^). The distribution of the molecular weight of polymer brushes was *M*_w_/*M*_n_ > 1.38.

##### BTO-*g*-PMMA (1.25)

A total of 0.75 g of initiator-functionalized barium titanate particles were dispersed in a mixture of 2.6 mL (25 mmol) MMA and 5.3 mL of anisole. Next, 28 µL of 20 mg/mL (2.5 µmol) CuBr_2_ in dimethylformamide, DMF, stock solution was added to the dispersion. The mixture was sonicated overnight using a Bransonic^®^ CPX1800H ultrasonic cleaner (frequency 40 kHz, thermostat off). Then, 1.3 µL (5.0 µmol) of Me_6_TREN and 0.8 mg (5.0 µmol) of AIBN were added to the dispersion. The mixture was degassed by nitrogen bubbling. It was then heated to 60 °C and stirred for 24 h. The product was isolated by precipitation in methanol. The molecular weight of the polymer was determined by size exclusion chromatography (SEC) after polymer cleavage from the particles.

##### BTO-*g*-PMMA (0.43) and BTO-*g*-PMMA (0.18)

A total of 1.0 g of barium titanate particles functionalized by the initiator were dispersed in a mixture of 2.6 mL (25 mmol) MMA and 2.6 mL anisole. Then, 18 µL of 20 mg/mL (1.6 µmol) CuBr_2_ in DMF stock solution was added to the dispersion. The mixture was sonicated overnight using a Bransonic^®^ CPX1800H ultrasonic cleaner (frequency 40 kHz, thermostat off). Then, 1.1 µL (4.0 µmol) of Me_6_TREN and 4 cm (BTO-*g*-PMMA (0.43)) or 1 cm (BTO-*g*-PMMA (0.18)) of Ø1 mm copper wire were added to the dispersion. The mixture was degassed by nitrogen bubbling. It was stirred at room temperature for 24 h. The product was isolated by precipitation in methanol and analyzed by SEC after cleavage from particles.

### 2.2. Methods of Hybrid Composites Characterization

Broadband dielectric spectroscopy measurements were performed in the frequency range from 5 × 10^−1^ to 3 × 10^6^ Hz and in the temperature range from −120 to 160 °C using a broadband dielectric spectrometer equipped with Quatro Cryosystem (Novocontrol Technologies GmbH & Co. KG, Germany).

The analysis of the experimental dielectric data was based on the Havriliak–Negami (HN) equation:

(1) $$\varepsilon^{*}\left(\omega \right)=\varepsilon_{\infty }+\frac{\Delta \varepsilon }{{\left[1+{\left(i\omega \tau \right)}^{\alpha_{\mathrm{NH}}}\right]}^{\beta_{\mathrm{NH}}}}$$

where ε* is complex dielectric permittivity, ω is an angular frequency, Δε is the dielectric relaxation strength defined as Δε = ε_s_ − ε_∞_ (ε_s_ and ε_∞_ are the low and the high-frequency limits of the real part ε’(ω) of the dielectric function ε*(ω)), τ is the characteristic relaxation time, whereas α_HN_ and β_HN_ are Havriliak–Negami (HN) parameters, that can be connected to the width and asymmetry of the loss peak, respectively. The dielectric loss spectra ε”(ω) were fitted using WinFit software (Novocontrol Technologies GmbH & Co. KG, Germany).

^1^H NMR spectra in liquid phase were obtained using an Avance II Plus instrument (700 MHz) spectrometer (Bruker, Billerica, MA, USA) with CDCl_3_ used as both internal standard with characteristic shift of δ = 7.26 ppm and solvent/dispersant. The ^1^H NMR measurements of the dispersions were made immediately after shaking. Solid state NMR experiments were performed using a 400 MHz NMR spectrometer equipped with a T3 MAS probe (Agilent Technologies, Inc, Santa Clara, CA, USA). Each sample was placed in a 4 mm diameter zirconia rotor and spun with an 8 kHz frequency. ^13^C spectra were recorded at 20 °C using a cross-polarization (CP) pulse sequence with phase modulated proton dipolar decoupling. The spin-lattice relaxation time T_1_ for protons was estimated using a magnetization recovery experiment with CP at 20 °C.

High-resolution transmission electron microscopy (HR-TEM) images were acquired using the ARF 200F electron microscope (JEOL Ltd. Tokyo, Japan) operated at 200 kV. TEM specimens were prepared by drop casting of diluted dispersion of BTO-*g*-PMMA systems in dichloromethane on copper grids.

Number-average molecular weights (*M*_n_) and molecular weight distributions (*M*_w_/*M*_n_) of PMMA grafted from the BTO surface were determined by size-exclusion chromatography (SEC, Waters Co., Milford, MA, USA). Prior to the SEC analysis, the samples were first dispersed in tetrahydrofuran and mixed with a few drops of hydrochloric acid. The solvent and the acid were removed under steady nitrogen flow. The detached polymer was extracted with tetrahydrofuran and filtered through a 450 nm PTFE filter. SEC was carried out with a Waters 515 pump and a Waters 410 differential refractometer with PSS columns (Styrogel 10^5^, 10^3^, and 10^2^ Å) in THF as an eluent at 35 °C and at a flow rate of 1 mL/min. Linear PMMA standards were used for calibration.

The absorption spectra in the middle IR region were collected with a Nicolet iS50 FT-IR spectrometer (Thermo Fisher Scientific, Waltham, MA, USA) equipped with an ATR accessory with a diamond crystal. Solid-state powder samples were used without any pretreatment. The spectral resolution was 2 cm^−1^, and 128 scans were averaged.

Raman measurements were made with the use of a Raman spectrometer T64000 (Jobin Yvon, HORIBA Ltd., Kyoto, Japan) equipped with an Olympus B-40 microscope (Olympus Corporation, Tokyo, Japan). Powder samples were measured without pretreatment. Laser wavelength and laser power were 514.5 nm and 15 mW, respectively. The acquisition was repeated 2 times for 30 s per measuring point.

Differential scanning calorimetry thermograms were recorded with the DSC 3 instrument (Mettler Toledo, Columbus, OH, USA). The samples were heated at a rate of 10 K/min with a 2 mL/min nitrogen gas purge in standard sealed aluminum crucibles. Measurements were made in a temperature range of 20 to 160 °C, and the results of the second heating run were analyzed. Temperature and heat flow were calibrated using indium and zinc melting point standards. Precise determination of the glass transition temperature (*T*_g_) of PMMA was difficult in nanocomposites. As expected, *T*_g_ of PMMA in BTO-*g*-PMMA systems overlapped with the phase transition between the tetragonal and cubic phases of BTO occurring at about 125 °C [30]. Thus, the *T*_g_ of PMMA has been reported herein as an onset point of the respective glass transition step determined according to the procedure shown in Figure S1 in Supporting Information (SI) and, additionally, as a range between onset and endset points (see Table 1).

Thermogravimetric analysis (TGA) was performed using the TGA1 STARe SYSTEM (Mettler Toledo, Columbus, OH, USA) in the temperature range from 25 to 900 °C. The heating rate was 10 °C/min, and the measurements were performed under argon flow of 50 mL/min up to 650 °C, and then continued under air flow with the same flow rate.

## 3. Results and Discussion

### 3.1. General Characterization of BTO-g-PMMA Nanomaterials

The general specifications of obtained core–shell BTO-*g*-PMMA composites are included in Table 1. The molecular weight of the PMMA chains grafted onto the BaTiO_3_ particles was similar to that of the linear PMMA used as reference material. That was needed for comparative studies of the dynamics of PMMA macromolecules in all systems. A tacticity of polymer chains in a reference PMMA sample and grafted on BTO nanoparticles was analyzed in liquid phase (in CDCl_3_ solution) by ^1^H NMR spectroscopy. The results, collected in Figure S2, reveal that the PMMA grafted from the surface of BaTiO_3_ by ATRP showed similar tacticity compared to the reference PMMA. In ^1^H NMR spectra, three proton signals of the methyl (–CH_3_) groups could be distinguished, corresponding to the three conformations of PMMA (isotactic, heterotactic, syndiotactic located at: δ = 1.27 ppm, δ = 1.04 ppm, and δ = 0.8 ppm, respectively). It should be noted that the syndiotactic conformation was predominant in all investigated samples, as demonstrated by the integration of signals, and increased from 0.6 to 0.7 for neat PMMA and BTO-*g*-PMMA (0.18), respectively. The inorganic content in the BTO-*g*-PMMA nanocomposites, namely 33%, 54%, or 62%, was determined based on TGA measurements (see Figure S3).

TEM images were performed to characterize the shape and surface of obtained nanocomposites. Images recorded at low magnification (Figure S4a) show that neat BTO particles used for modification have an average diameter of about 200 nm. These ceramic nanoparticles are not perfectly spherical; therefore, the calculated nanoparticles’ surface and, consequently, grafting density of tethered chains may slightly differ from the real one. In Figure S4b, images collected at high magnification can be seen where two regions of different contrasts can be observed for the BTO-*g*-PMMA nanocomposites. The thickness of the organic layer, seen as a brighter or shadow area on a ceramic surface, can be estimated to be equal to about 5 nm. The HR-TEM results confirmed the core–shell structure in investigated hybrid nanomaterials.

The FTIR measurements (see Figure S5) showed consistent results with thermal analysis, as described in Section 3.2.3 (Intermolecular interactions and conformational changes). It is important to note that in a case of lower PMMA grafting density, the content of BTO was higher, as expected, considering a comparable average molecular weight of PMMA chains in all systems.

The DSC thermograms of the investigated samples, presented in Figure S1 in SI, show only a single glass transition in a range of 110–150 °C related to PMMA [31,32,33]. The glass transition temperatures for all samples are collected in Table 1. The *T_g_* of PMMA increases with an increase in the inorganic content, which may be related to the effective immobilization of the PMMA chains on the BTO surface, as well as its stronger effect in the case of very low grafting density. Similar findings were reported in the case of other organic–inorganic systems in which increased glass transition temperature was interpreted as a consequence of the increased steric hindrance in polymer-grafted particles that counteracts the relaxation of surface-grafted polymer chains [34,35,36].

The characteristic bands of tetragonal BTO, i.e., peaks at 305, 512, and 715 cm^−1^ dominate the Raman spectra of the BTO-*g*-PMMA samples, as Figure S6 shows [13,14,15]. High similarity between the Raman spectrum of bare BTO nanoparticles and those after polymer grafting proved that the ATRP process and further procedures did not influence the structure of the inorganic phase.

### 3.2. Molecular Dynamics in BTO-g-PMMA Nanocomposites

#### 3.2.1. Broadband Dielectric Spectroscopy

**Figure 1:**
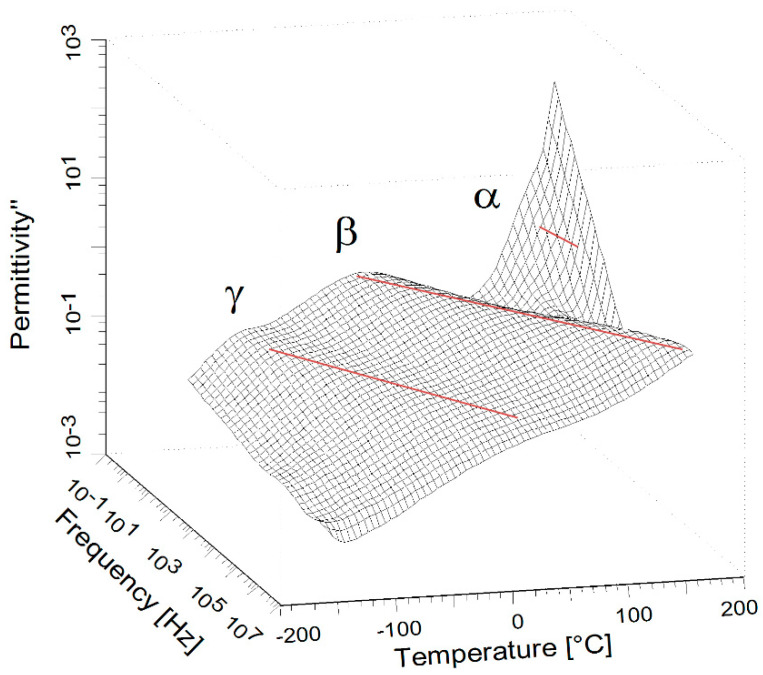
3D map (frequency–temperature dependences of dielectric loss (ε”)) for BTO-*g*-PMMA (0.43). The solid red lines, drawn as a guide for eyes only, indicate relaxation processes.

Figure 1 shows an exemplary BDS measurement performed over a broad frequency–temperature range for the investigated core–shell systems. Three relaxation processes detected in BTO-*g*-PMMA samples are similar to those characteristics of neat PMMA.

At the highest temperature, the α-relaxation is partially hidden under the DC conductivity shoulder. This cooperative relaxational process is associated with the dynamic glass transition temperature. At lower temperatures, the local molecular processes are visible, namely β and γ-relaxations. The β process is very pronounced in PMMA and is assigned to rotational or conformational changes of the ester moiety (–COOCH_3_) around the bond linked with the main chain, while the γ-relaxation is due to the rotation of the α-methyl group bonded to the main chain or humidity [37].

**Figure 2:**
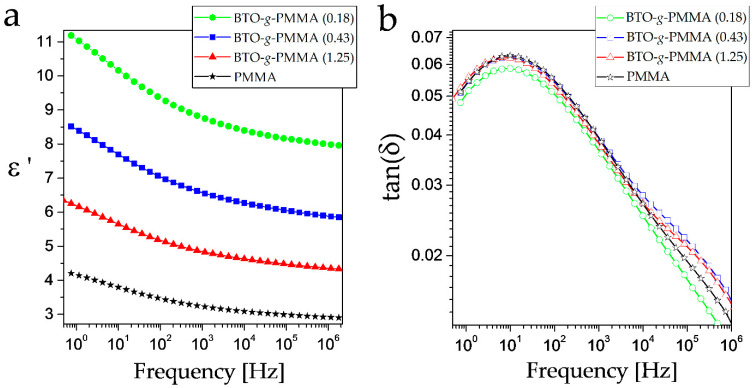
Frequency dependence of real part of dielectric permittivity (**a**) and loss tangent (**b**) for BTO-*g*-PMMA nanocomposites determined at 20 °C.

Increasing the BTO content in composites results in an increase in the real part of dielectric permittivity (ε’) called the dielectric constant. It allows classifying the obtained systems as materials with elevated values of dielectric permittivity. At 1 MHz at 20 °C, the ε’ are equal: 4.4, 5.9, and 8.0 for composites containing 33, 54, and 62 wt.% of BTO, respectively (Figure 2a). These values correspond to 9, 19, and 24% of the BTO volume fraction in the studied systems. Note that tetragonal BTO in the form of single crystals or micrometer-sized particles is a ferroelectric material with a dielectric constant of a few thousand at room temperature [38,39], while BTO nanocrystals are a high-k inorganic material [40]. The observed increase in ε’ is in good agreement with the Lichtenecker rule (see Table S1 in SI), according to which the effective dielectric permittivity of a composite is a function of the dielectric permittivity of the polymeric matrix and the inorganic filler, as well as its volume fractions [41]. Figure 2b shows the dielectric spectra of the samples studied in loss tangent representation at 20 °C. At this temperature, a significant β relaxation process occurs with a maximum at about 10 Hz, whereas the weaker γ process is partially hidden and appears as a high-frequency shoulder with a maximum at c.a. 105 Hz. The tan(δ), being equal to tan(δ) = ε”/ε’, slightly increases (similarly to ε’) with an increase in BTO content in the composites throughout the investigated frequency range; however, the differences are very small.

These results are in agreement with the findings of Xie et al. [42], who also observed an increase in dielectric permittivity with a higher inorganic contribution with no significant changes in tan (δ).

A detailed analysis was performed to assess the core–shell composite organization on local PMMA motions. The β process is the most prominent relaxation detected in PMMA, which was observed in all investigated materials.

**Figure 3:**
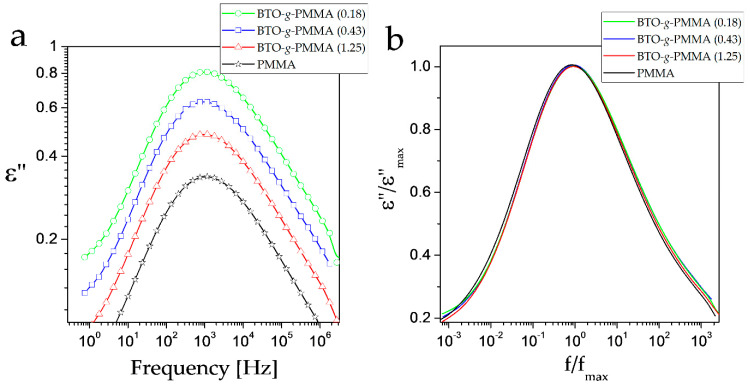
Frequency dependence of imaginary part of dielectric permittivity (**a**) and its normalized curves (**b**) for BTO-*g*-PMMA core–shell composites collected at 80 °C.

In Figure 3, the plots of frequency dependence of ε” for BTO-*g*-PMMA core–shell composites determined at 80 °C are shown. At this temperature, the dielectric loss (ε”) spectra for β relaxation are located symmetrically in the investigated frequency window and are only weakly affected by other relaxations. An increase in dielectric relaxation strength, Δε, with an increase in BTO content in composites, is clearly visible in Figure 3a; see also Table 2. Analysis of the β processes in the normalized representation of ε”/ε”_max_, presented in Figure 3b, showed tiny changes in the symmetry of the relaxation time distribution of these processes in the core–shell composites. These changes are reflected in β_HN_ parameters (Table 2). Maximum β relaxation is located at a similar position, i.e., having similar τ_max_ values, whereas average relaxation time τ is affected by the higher asymmetry of this process in composites. This asymmetry may originate from a different local electric field that ester moieties responsible for the β process are subjected in composites as compared to neat PMMA. These findings are in agreement with the broadening of NMR lines in solid-state investigations (discussed later) and with the increase in *T*_g_ in nanohybrid systems compared to neat PMMA, as seen by DSC thermograms. The observed behavior can originate from (i) stiffening of PMMA chains in systems with BTO or (ii) greater heterogeneity in the context of chemical environments (the range of chemical shifts in NMR spectra for a given nucleus for nanohybrids is expanding due to the presence of additional, slightly different neighbors, e.g., BTO nanoparticles in comparison with neat polymer).

**Figure 4:**
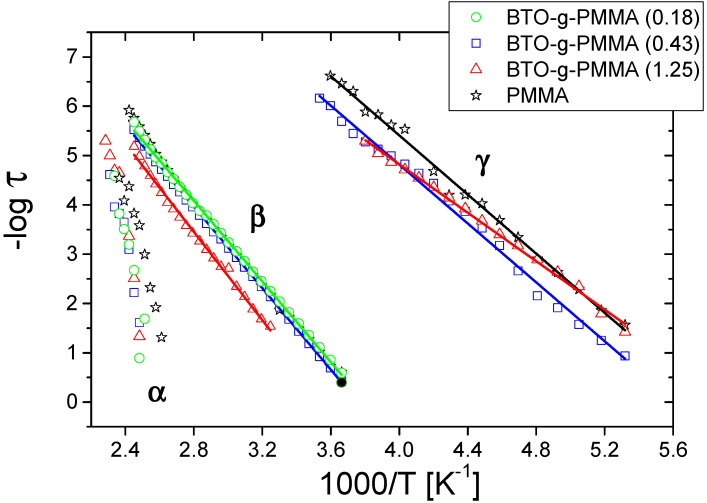
Arrhenius plot for the core–shell BTO-*g*-PMMA composites performed from BDS experiments. Data for PMMA were added for comparison.

Based on BDS spectra, the activation map was constructed, as seen in Figure 4. Secondary relaxation processes found in the investigated materials, γ and β, relate to local motions in the glassy state and have non-cooperative nature, so that they obey the Arrhenius equation:

(2) $$\tau =\tau_{0}\exp\left(E_{A}/k_{B}T\right)$$

where τ—relaxation time, E_A_—activation energy or energy barrier for the investigated relaxation, τ_0_—the pre-exponential factor, k_B_—Boltzmann constant, and T—temperature [K].

Contrary to secondary processes, α-relaxation is associated with cooperative motions (in a range of glass transition temperature) and can be described by the Vogel-Fulcher-Tammann (VFT) equation:

(3) $$\tau =\tau_{0\alpha }\left[{\mathrm{DT}}_{0}/\left(T-T_{0}\right)\right]$$

where τ_0α_ is a preexponential factor, T_0_ is the so-called Vogel temperature (representing ideal glass transition temperature), which is generally 30÷70 K below T_g_ [43] and D is the so-called strength parameter [44].

In investigated composites, α-relaxation is hidden under DC conductivity shoulder so that it is extracted by the fitting procedure. In Figure S7 (SI), exemplary results of fitting for segmental relaxation are shown. An applied fit procedure allowed estimating the segmental relaxation times of the investigated systems, which were measured at different temperatures. The obtained data are presented in Figure 4. It is seen that α relaxation for composites is shifted to higher temperatures as compared to the neat PMMA. This indicates a slowdown of the segmental dynamics resulting from the stiffening of the system. These findings agree with the DSC results shown in Figure S1 and discussed above. Due to the fact that the α-relaxation in the studied nanocomposites is hidden by other processes (DC and β-relaxation), further analysis of related parameters like Vogel temperatures or strength parameters and related with them fragility indexes, that reflex cooperativity of the systems and their correlation with the degree of intermolecular coupling of polymer chains, could have a large dose of uncertainty.

The activation energies for the detected secondary relaxations are collected in Table 3.

The activation energy for β-relaxation for neat PMMA was equal to 81.9 kJ/mol, while in the case of the grafted system, this energy is higher, and in the case of a high grafting density, it is equal to 85.7 kJ/mol for BTO-*g*-PMMA (1.25) and decreases to 78.3 kJ/mol for BTO-*g*-PMMA (0.18). Xie et al. [42] found a similar trend of E_A_ changes in BTO-PMMA nanocomposites; however, these authors claimed that BTO nanoparticles have no influence on the activation energy of β-relaxation. In our opinion, this comes from different dipolar interactions within carbonyl groups of PMMA and different polymeric chain organization of long PMMA chains on the BTO surface. In the case of low grafting density, the activation energy for relaxation β decreases as compared to the E_A_ determined for the neat PMMA. It means that dipole–dipole interactions of carbonyl groups PMMA are weakened probably due to competitive interaction of carbonyl/ester moieties with the accessible surface of the ceramics. The increase in the grafting density is connected with a denser packing of the tethered chains on the ceramic surface, which results in their different spatial organization (transformation into brush-like conformation), causing a strengthening of the dipolar interactions between the polymeric chains and an increase in E_A_ for β relaxation. This will be discussed in detail in a section devoted to vibrational spectra analysis.

Calculated E_A_ values for relaxation γ do not show an unequivocal effect of PMMA grafting from the BTO surface. This may be connected to the fact that this relaxational process is related to the local movement of the small structural moiety of PMMA structure, which is not sensitive to changes in polymer chain conformation [45]). Additionally, the dielectric strength of this relaxation is very small and decreases with a decrease in PMMA content in core–shell composites, causing a higher error in the estimation of E_A_ of γ process and even cannot be detected in the sample of high wt. % of BTO.

#### 3.2.2. Solid-State NMR

**Figure 5:**
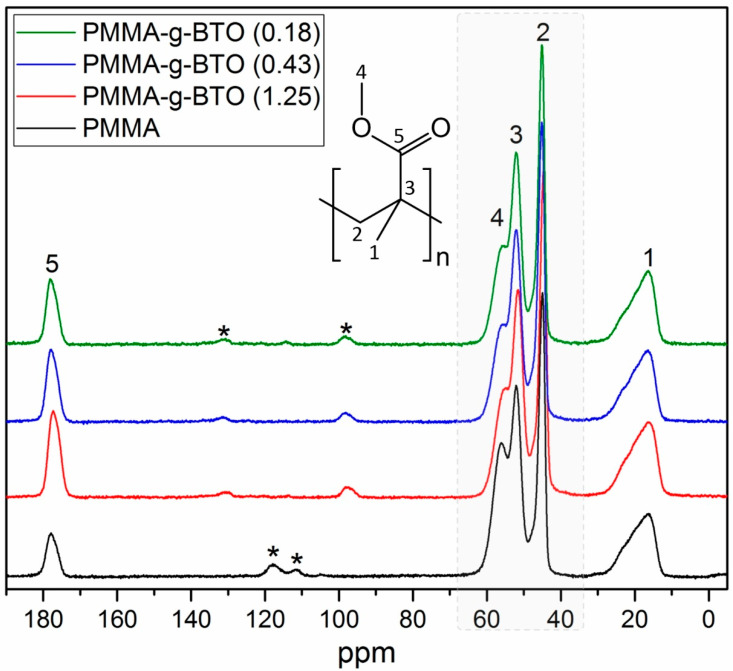
Comparison of the ^13^C CP/MAS NMR spectra recorded for BTO-*g*-PMMA (0.18)—(green), BTO-*g*-PMMA (0.43)—(blue), BTO-*g*-PMMA (1.25)—(red) and neat PMMA—(black). Signals marked with asterisks indicate rotational sidebands.

The ^13^C NMR spectra presented in Figure 5 confirm the presence of PMMA in all investigated composites. Five characteristic carbon signals [19,20] can be assigned as follows: (i) peak 1 at 17 ppm is related to the methyl group, (ii) peak 2 at 45 ppm is related to the methylene group, (iii) peak 3 at 52 ppm is related to the quaternary carbon of the polymeric chain, (iv) peak 4 at 56 ppm is related to the methoxy group, and (v) peak 5 at 177 ppm is related to carbonyl carbon. All the carbon signals observed in the case of hybrid materials display virtually the same chemical shifts as in the case of neat PMMA. Thus, the core–shell structure does not alter the average chemical shielding in the case of grafted polymer. Nevertheless, a deeper analysis of the NMR spectra reveals a quite significant change in the line shape, which seems to be a direct manifestation of polymer–nanoparticle interactions. To clearly display these differences between neat and grafted polymers, the spectra were analyzed in the most representative chemical shift range (marked gray in Figure 5), which corresponds to the carbons of the polymer backbone.

**Figure 6:**
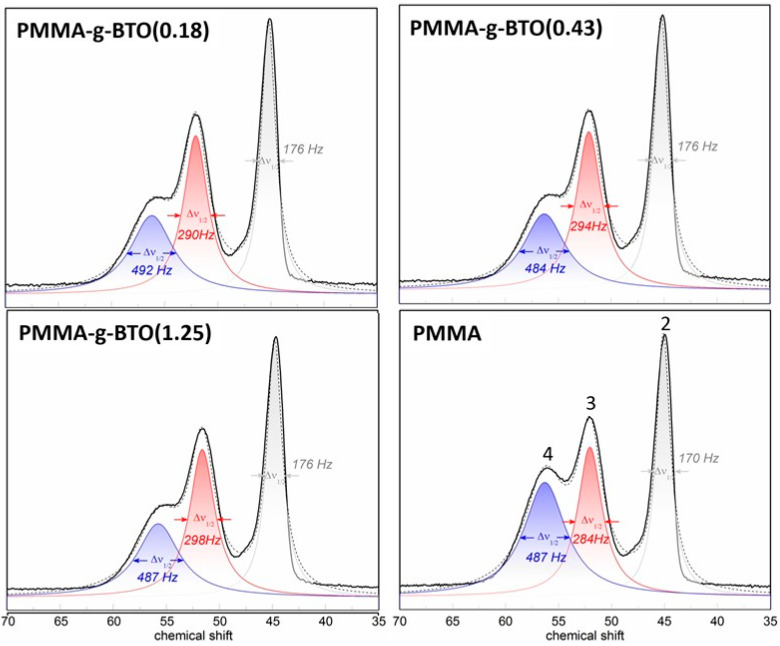
Deconvoluted NMR data and individual Lorentzian components half-width analysis.

The chemical shifts region marked with a gray dotted line is magnified and analyzed in Figure 5. It illustrates the chemical shift range, including two important NMR signals, both representing the polymer backbone, i.e., the methylene carbons and the quaternary carbons. The overall NMR line shape can be satisfactorily reproduced by the three independent Lorentzian components; see dotted lines. Such an approach enables precise assessment of the line shape evolution and monitoring of individual signals separately. Accordingly, the presented fits indicate that there is a systematic NMR line broadening observed in the case of the backbone signals from the grafted polymers with respect to the same signals observed in the case of neat PMMA. Although this effect is not substantial (the increase reaches ~3.5% in the case of the methylene carbon signals and ~4.9% in the case of quaternary carbons), it is worth emphasizing that it manifests consistently for all core–shell structures. Similar effects have already been reported in the case of other core–shell structures, such as nanodiamonds covalently linked to polyallylamine hydrochloride [46], dodecanethiolate-protected Au clusters [47], Au nanoparticles with octadecanethiolate coating [48], and other Au NPs monolayer ligand shells [49]. According to Marbella et al. [50], the NMR line broadening arises from the ‘solid-like’ environment introduced by the adjacent nanoparticle, leading to a reintroduction of chemical shift anisotropy (CSA) and stronger dipole–dipole coupling spin interactions. Hostetler et al. [47] suggest similar factors that appear to contribute to spectral broadening, namely (i) methylene groups closest to the surface of the NPs experience fast spin relaxation, (ii) various binding sites on the surface of the NPs lead to the distribution of chemical shifts. The mentioned arguments are in agreement with the data presented in Figure 6 that indicate an effective stiffening of the polymer chains grafted to the NPs. The observable NMR line broadening is a consequence of a faster spin relaxation (shorter spin–spin T_2_ relaxation time), directly suggesting the segmental dynamics slowdown. This finding is in good agreement with the BDS and DSC data shown above. The results of both experiments correspond well to the NMR data and together strongly support the idea that the overall polymer chain dynamics are restricted due to interactions with the solid, inorganic NPs surface.

Spin-lattice relaxation time measurements were done much below (more than 100 °C) the glass transition temperature, and hence, one can assume that we are far from the T_1_ (T) minimum, which is expected above *T*_g_. Therefore, similar relaxation times T_1_ observed in the case of neat PMMA, BTO-*g*-PMMA (0.43), and BTO-*g*-PMMA (0.18) are not surprising (see Figure S8). Interestingly, only the BTO-*g*-PMMA (1.25) sample differs substantially from the other samples and reveals a longer relaxation time T_1_. This result may be correlated with a much higher grafting density in the case of BTO-*g*-PMMA (1.25) with respect to the other two hybrid samples.

#### 3.2.3. Intermolecular Interactions and Conformational Changes

**Figure 7:**
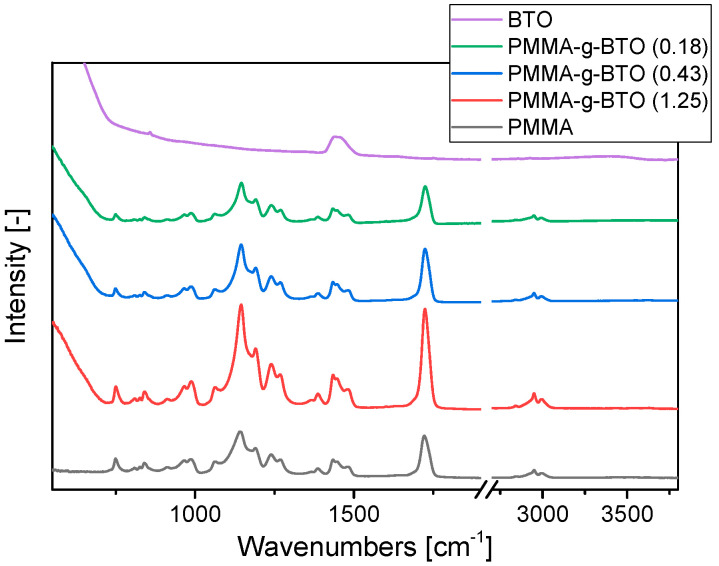
ATR FTIR spectra of BaTiO_3_, PMMA, and PMMA-*g*-BTO composites.

The ATR FT-IR spectra of the reference materials (neat PMMA and the BTO) and PMMA-*g*-BTO nanohybrids with a 200 nm tetragonal core in the middle IR region are shown in Figure 7. The CH_2_/CH_3_ stretching modes at 2994 cm^−1^, 2950 cm^−1^, 2842 cm^−1^, C=O stretch at 1722 cm^−1^ and the asymmetric C–O–C stretch at 1192 cm^−1^ and 1141 cm^−1^ are visible for hybrid particles as well as for neat PMMA [51]. The presence of these bands indicates that PMMA was successfully grafted from BaTiO_3_. There are only two prominent adsorption bands for bare BTO at 1441 cm^−1^ and 855 cm^−1^ which represent the stretching vibration of –CO_3_^2−^ and CO^2−^ from the residual BaCO_3_ in the BTO [52].

**Figure 8:**
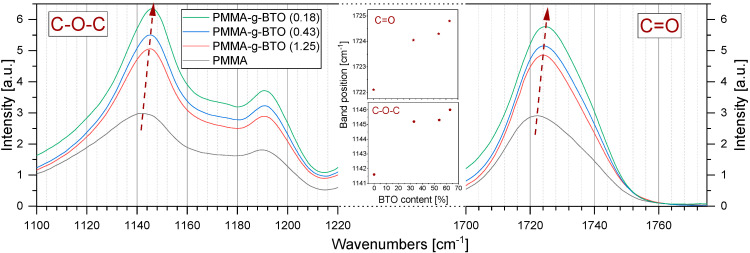
ATR FT-IR spectra of PMMA-*g*-BTO nanocomposites at the regions of C=O and C–O–C stretching vibrations. All spectra were normalized to the intensity (signal amplitude) of the line with a maximum at 750 cm^−1^. Inset in the middle part of the chart shows changes in the positions of the characteristic line maxima vs. BTO percentage.

Most of the absorbance bands are located at the same wavenumbers independently of the sample. Only for three bands, significant changes were found. Bands relating to C=O stretching (1722 cm^−1^) and C–O–C stretching (1141 cm^−1^) shift toward higher wavenumbers with increasing BTO content (see Figure 8), while the intensity of the line assigned to CO^2−^ vibration (855 cm^−1^) increases with increasing BTO content. (Figure S5).

As Figure 8 shows, the peak maximum characteristic for C=O stretching vibrations evidently shifts from 1722 cm^−1^ for neat PMMA to 1725 cm^−1^ for the sample with the highest BTO content. This effect may be explained by the C=O bond shortening, resulting from the weakening of interchain interactions between PMMA chains [53]. Considering the relatively high molecular weight of grafted PMMA chains and a reverse relation between the grafting density and BTO content in the sample, one can expect different organization of PMMA macromolecules on the BTO surface. The high molecular weight and low grafting density (high BTO content in the sample) result in relatively large surface areas of BTO accessible for PMMA chains. To reduce the high surface energy of BTO nanocrystals, one can expect that the PMMA chain covers the surface and is attached to it by physical interactions (dipole–dipole and/or van der Waals forces). The increase in PMMA grafting density results in denser packing of polymer chains and stronger interactions between them manifested in C=O stretching mode shift to lower wavenumber. These findings are in good agreement with the changes in E_A_ changes of β-relaxation in neat PMMA and PMMA-*g*-BTO composites calculated from BDS measurements.

Analogous noticeable differences were also observed in the region of C–O–C stretching vibrations (Figure 8). An increase in inorganic content in the sample (lower grafting density) results in a shift of the C–O–C absorption band from 1142 cm^−1^ for neat PMMA to 1146 cm^−1^ for the sample with the highest BTO content. This effect may be related to conformational changes in the ester group or shortening of the C–O bond between the methyl group carbon and oxygen with simultaneous lengthening of the C–O bonds between carbonyl carbon and oxygen. Changes in C–O–C bond lengths may be caused by interactions of C=O groups of ester groups with the environment, as shown previously for poly(oligoether methacrylates) [54]. Observed changes in the position of bands related to the vibrations of the ester group prove that the PMMA-PMMA and PMMA-BTO interactions depend on the grafting density.

However, the Raman spectra of BTO-*g*-PMMA samples are dominated by BTO bands; some peaks characteristic of PMMA are also visible (full spectra are shown in Figure S6). At 812 cm^−1^, the C–O and C–C stretching modes are manifested, at 1450 cm^−1^, the bending of CH_2_ and CH_3_, while at 2953 and 3003 cm^−1^ symmetric and asymmetric stretching of the -CH_x_ groups) [55,56]. The bands can be observed for neat PMMA and PMMA-*g*-BTO samples clearly at the similar wavenumbers.

**Figure 9:**
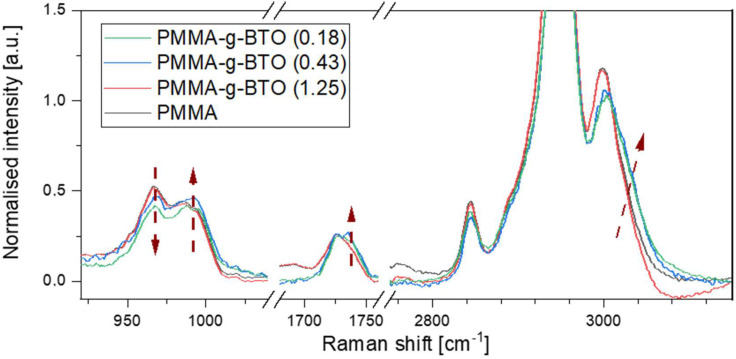
Raman spectra of BTO-*g*-PMMA nanocomposites at some selected regions.

Figure 9 shows some selected Raman bands of PMMA sensitive to the presence of BTO nanocrystals. In the region related to carbonyl group vibrations (1700–1750 cm^−1^), at least two components are visible, and their relative intensities evidently depend on the BTO content. The higher the BTO content, the more intense the line at higher wavenumbers. This result is consistent with the FTIR results and proves that the intermolecular interactions in the BTO-*g*-PMMA nanohybrids depend on the composition and accessibility of the BTO surface for PMMA chains.

In the region sensitive to conformational changes and related to coupling C-C stretching vibrations, also called skeletal modes, again, at least two components are easily distinguished, proving that the chain conformation of grafted PMMA chains depends on the grafting density. This is expected if the accessibility of the BTO surface is considered. As mentioned above, if the grafting density is low, the PMMA chains have enough space to curl and entangle, forming mushroom-like structures [18,57]. Increasing grafting density results in higher extension of PMMA chains, which may also result in their higher stiffness manifested by other techniques. Similar differences in PMMA spectra were observed by Blaszczyk–Lezak et al. [58], who studied PMMA nanofibers formed in confined spaces. They showed that the relative intensity of the 978 cm^−1^ line decreases if the PMMA is strongly confined. This is consistent with the results present in this work.

Additionally, changes in stretching vibrations of -CH_3_ groups (3005 cm^−1^) are well visible in the high-frequency range of the Raman spectrum. With increasing BTO content, the band corresponding to CH_3_ groups shifts to higher wavenumbers. This change may be explained by two reasons, (i) the line shift results from conformational changes of PMMA chains, or (ii) the line shift corresponds to the charge redistribution within the ester group resulting from changes in intermolecular interactions with the environment (such changes were previously reported for some polymer dispersed in water [59]). Univocal answer to the problem is very difficult, as both –CH_3_ groups contribute to this band. Moreover, both conformational changes (manifested by the shift of lines corresponding to skeletal modes), as well as changes in intermolecular interactions (visible in the shift of bands related to carbonyl groups), occur simultaneously.

## 4. Conclusions

The nanocomposites containing barium titanate and poly(methyl methacrylate) grafted from this ceramic surface were synthesized and investigated to determine the role of the inorganic surface on the molecular dynamics of polymer chains, their structure, and interactions. The obtained composites have a core–shell organization, confirmed by HR-TEM investigations, and exhibit elevated dielectric permittivity, as compared to neat PMMA.

To enhance the effect of the inorganic surface on the behavior of PMMA chains, the grafting density was increased from 0.18 to 0.43 and to 1.25 chains/nm^2^. The syndiotactic conformation of PMMA was predominant in all investigated composites as well as in neat PMMA, and its fraction did not change significantly with varied grafting density.

It was found that the *T*_g_ of PMMA in the investigated composites increased with increasing content of the inorganic phase, regarding the grafting density. These findings are in good agreement with results obtained by BDS and ^13^C NMR techniques. The BDS studies showed that the α relaxation for composites was shifted to higher temperatures/lower frequencies as compared to neat PMMA. This indicates a slowdown of the segmental dynamics resulting from the stiffening of the system. In ^13^C NMR studies, the line broadening for carbons located in the PMMA backbone was observed for nanocomposites as compared to neat PMMA. This came from the ‘solid-like’ environment introduced by the adjacent BTO nanoparticles.

The grafting density also affected the interactions between polymer chains. This was proved by careful analysis of FT-IR spectra and local dynamics, namely the β-process seen by BDS. The increase in PMMA grafting density resulted in denser packing of polymer chains and stronger interactions within polymer chains as manifested in the C=O stretching mode shift toward lower wavenumbers. These findings are in good agreement with the changes in activation energy of β-relaxation in PMMA-*g*-BTO composites calculated from BDS measurements.

The experimental techniques used for the study of BTO-*g*-PMMA nanocomposites allow a broad characterization of the structure and molecular dynamics at different scales as well as the ceramic–polymer and polymer–polymer interactions. A better understanding of the structure–dynamic relationship in core–shell hybrid systems can be helpful for the prediction and design of new dielectric materials with targeted properties.

## Figures and Tables

**Table 1:** Sample specification of PMMA and core–shell BTO-*g*-PMMA composites based on barium titanate core of 200 nm size; (*f*_inorg_ = percentage of inorganic component).

Sample Name	PMMA *M_n_*	PMMA *M_w_*/*M_n_*	Grafting Density (chain/nm^2^)	*f*(inorg)	*T*_g_ (°C) Range	
					*Onset Point*	*Endset Point*
PMMA	3.50 × 10^5^	1.17	-	0%	91	137
BTO-*g*-PMMA (1.25)	1.52 × 10^5^	1.77	1.25	33%	103.5	144
BTO-*g*-PMMA (0.43)	1.85 × 10^5^	2.38	0.43	54%	109	142
BTO-*g*-PMMA (0.18)	3.18 × 10^5^	1.38	0.18	62%	111	142

**Table 2:** Havriliak–Negami (HN) parameters for β-relaxation processes in studied BTO-*g*-PMMA composites obtained from analysis of dielectric spectra at 80 °C. The data for neat PMMA was added for comparison.

Sample	τ (s)	τ_max_ (s)	Δε	α_HN_	β_HN_
PMMA	4.9 × 10^−4^	1.4 × 10^−4^	2.13	0.52	0.51
BTO-*g*-PMMA (1.25)	5.4 × 10^−4^	1.6 × 10^−4^	2.72	0.53	0.51
BTO-*g*-PMMA (0.43)	6.4 × 10^−4^	1.7 × 10^−4^	3.83	0.53	0.47
BTO-*g*-PMMA (0.18)	5.7 × 10^−4^	1.5 × 10^−4^	4.87	0.53	0.48

**Table 3:** Activation energies of local relaxations calculated for neat PMMA and BTO-*g*-PMMA core–shell composites.

Sample’s Name	E_A_ of β Relaxation [kJ/mol]	*Ε*_A_ of γ Relaxation [kJ/mol] ± 2
PMMA	81.9 ± 0.9	57
BTO-*g*-PMMA (1.25)	85.7 ± 1.3	47
BTO-*g*-PMMA (0.43)	79.1 ± 0.4	57
BTO-*g*-PMMA (0.18)	78.3 ± 0.6	γ not visible

## Data Availability

Not applicable.

## Supplementary Materials

The following supporting information can be downloaded at: https://www.mdpi.com/article/10.3390/molecules27196372/s1, Figure S1. (a) The first derivative method for the determination of the glass transition temperature from DSC thermograms—exemplary results for neat PMMA (top graph), and (b) exemplary second DSC scans collected for neat PMMA, neat BTO, and BTO-*g*-PMMA (1.25) sample. Insets in bottom chart show the dependences of glass transition temperature of PMMA (onset value) as a function of BTO content and grafting density. (c–e) The results of the first derivative analysis of DSC thermograms for studied nanocomposites. Figure S2. ^1^H NMR spectra of PMMA and BTO-*g*-PMMA composites in liquid phase obtained using an Avance II Plus instrument (700 MHz), Bruker (Billerica, USA). Figure S3. TGA curves for linear PMMA and BTO-*g*-PMMA composites. Figure S4. HR-TEM images for BTO-*g*-PMMA composites with different grafting densities collected with lower (Figure S4a) and higher magnification (Figure S4b), respectively. Figure S5. ATR FT-IR spectra of BTO-*g*-PMMA nanocomposites at the region where the vibrations of BTO are expected (c.a. 855 cm^−1^). Inset shows the dependency of the intensity at 855 cm^−1^ vs. BTO percentage. Figure S6. Raman spectra of BTO, PMMA, and BTO-*g*-PMMA hybrid nanocomposites. Figure S7. Exemplary results for fitting of BDS spectra collected at 120 °C with the use of WinFit software for (a) neat PMMA and (b) BTO-*g*-PMMA (0.43) composite. Figure S8. Spin-lattice relaxation time T1 measurements. ^13^C CP/MAS NMR spectra recorded at 20 °C for the investigated systems (a); Magnetization recovery for protons measured using the cross-polarization method at 20 °C for two individual signals attributed to methylene carbons 2 (b), and quaternary carbons 3 (c). Table S1. Sample specification of core–shell BTO-*g*-PMMA composites. Calculated values of ε for composites, according to the Lichtenecker rule, were obtained taking into account BTO density equal to 6.02 g/cm^3^ and its ε = 180.

## Sample Availability

Samples of the compounds are available from the authors.
