# Supplementary material for: Molecular Dynamics and Structure of Poly(Methyl Methacrylate) Chains Grafted from Barium Titanate Nanoparticles
Source: Molecules. 2022 Sep 27;27(19):6372. doi: 10.3390/molecules27196372 (PMC9571223; doi:10.3390/molecules27196372)
Supplement: Supplementary file 1 [file molecules-27-06372-s001.zip › molecules-1868992-supplementary.pdf]

molecules

*Supporting Information*

# Molecular Dynamics and Structure of Poly(Methyl Methacrylate) Chains Grafted from Barium Titanate Nanoparticles

**Aleksandra Wypych-Puszkarz[1*], Onur Cetinkaya[1, 2], Jiajun Yan[3], Ruslana Udovytska[1], Jarosław Jung[1], Jacek Jenczyk[4], Grzegorz Nowaczyk[4], Stefan Jurga[4,†], Jacek Ulański[1], Krzysztof Matyjaszewski[1,3], Joanna Pietrasik[5] and Marcin Kozanecki[1]**

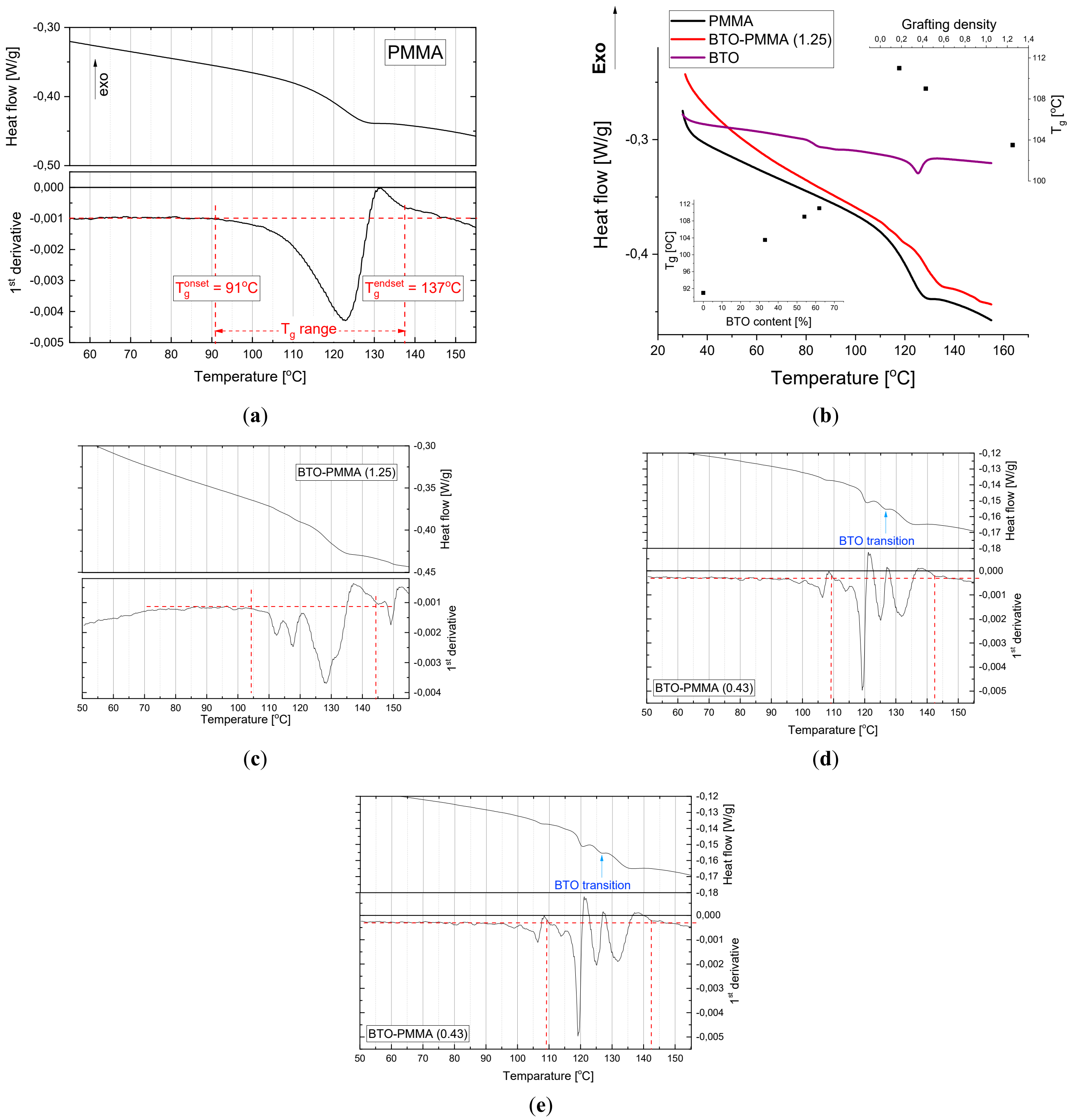


**Figure S1.** (**a**) The first derivative method for the determination of the glass transition temperature from DSC thermograms – exemplary results for neat PMMA (top graph), and (**b**) exemplary second DSC scans collected for neat PMMA, neat BTO, and BTO-*g*-PMMA (1.25) sample. Insets in bottom chart show the dependences of glass transition temperature of PMMA (onset value) as a function of BTO content and grafting density. (**c**–**e**) The results of first derivative analysis of DSC thermograms for studied nanocomposites.

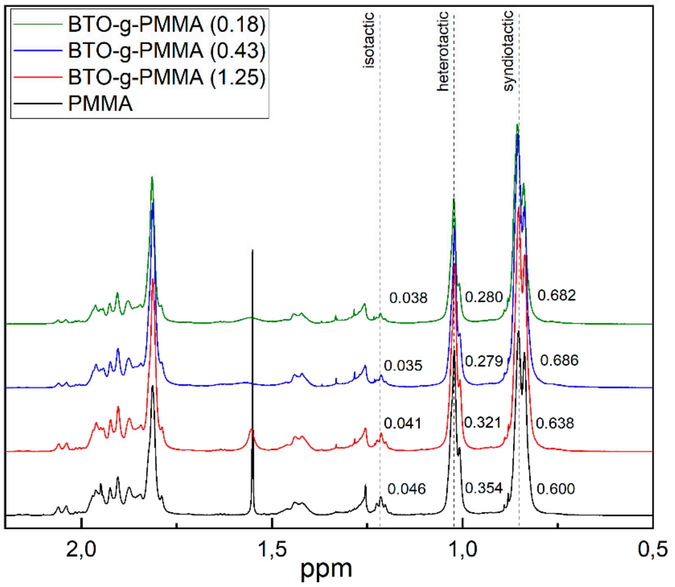


**Figure S2.** $^{1}$H NMR spectra of PMMA and BTO-*g*-PMMA composites in liquid phase obtained using an Avance II Plus instrument (700 MHz), Bruker (Billerica, USA).

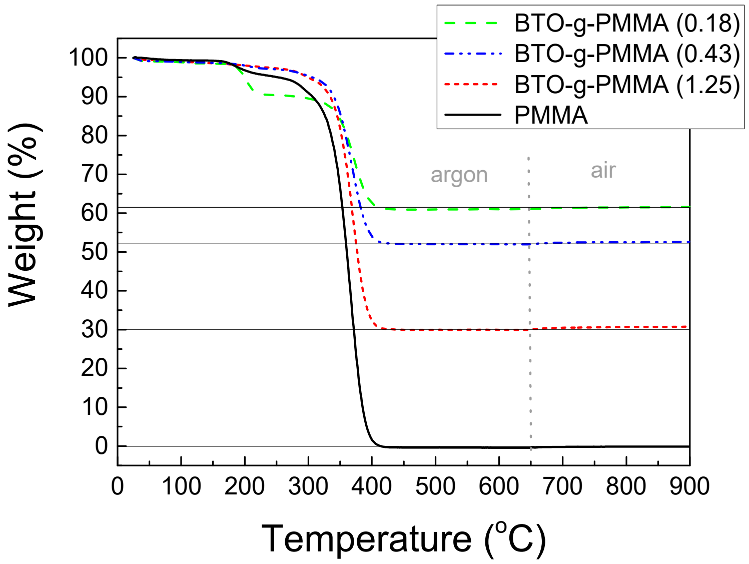


**Figure S3.** TGA curves for linear PMMA and BTO-*g*-PMMA composites.

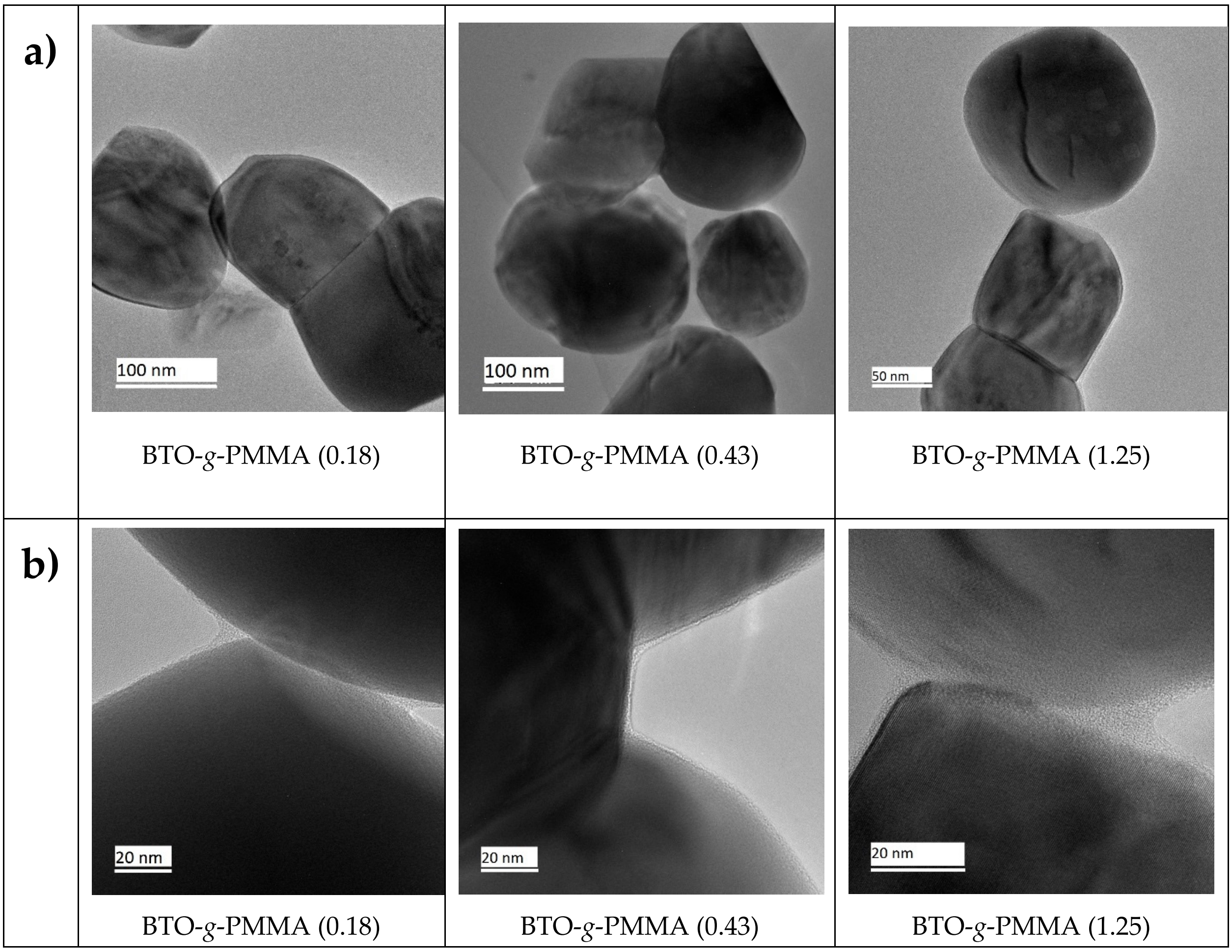


**Figure S4.** HR-TEM images for BTO-*g*-PMMA composites with different grafting densities collected with lower (Figure S2 a) and higher magnification (Figure S2 b), respectively.

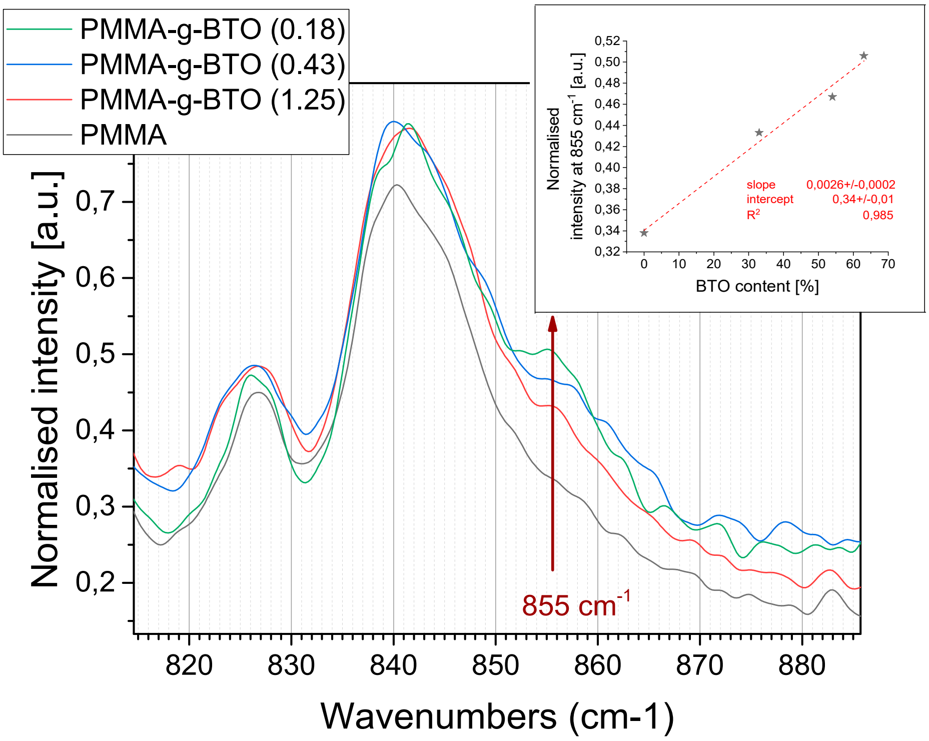


**Figure S5.** ATR FT-IR spectra of BTO-*g*-PMMA nanocomposites at the region where the vibrations of BTO are expected (c.a. 855 cm$^{-1}$). Inset shows the dependency of the intensity at 855 cm$^{-1}$ vs. BTO percentage.

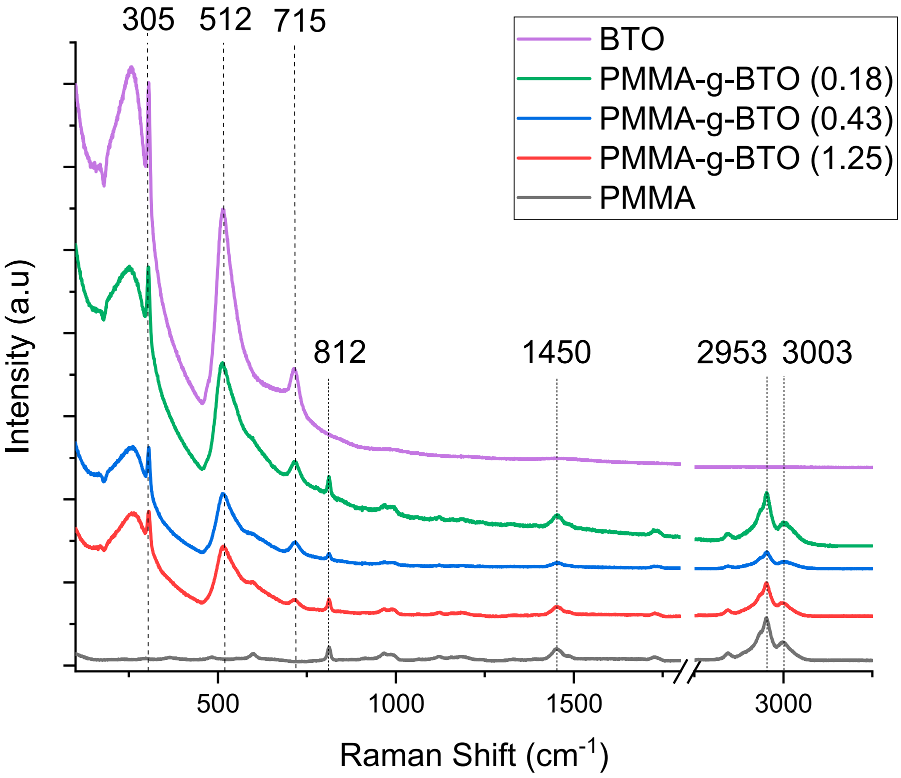


**Figure S6.** Raman spectra of BTO, PMMA and BTO-*g*-PMMA hybrid nanocomposites.

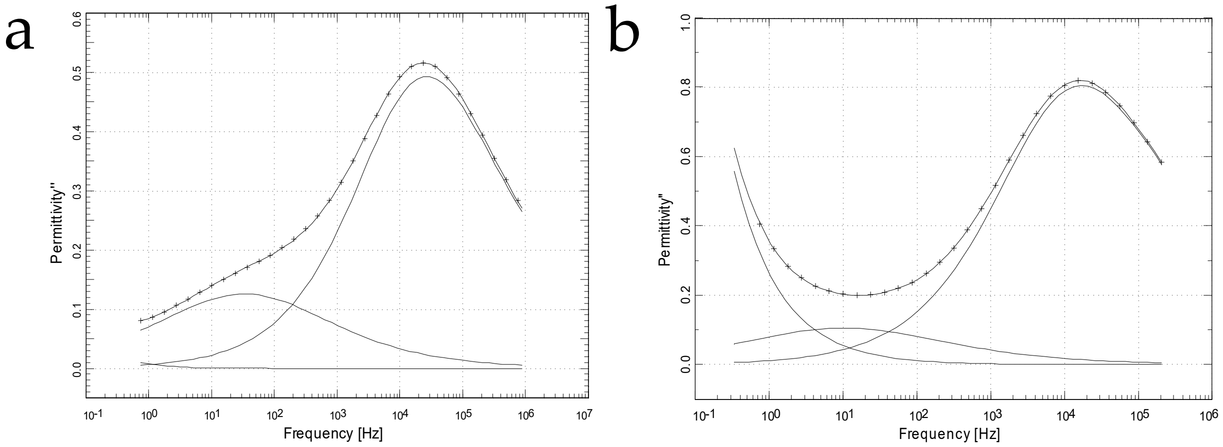


**Figure S7.** Exemplary results for fitting of BDS spectra collected at 120 °C with use of WinFit software for (**a**) neat PMMA, and (**b**) BTO-*g*-PMMA (0.43) composite.

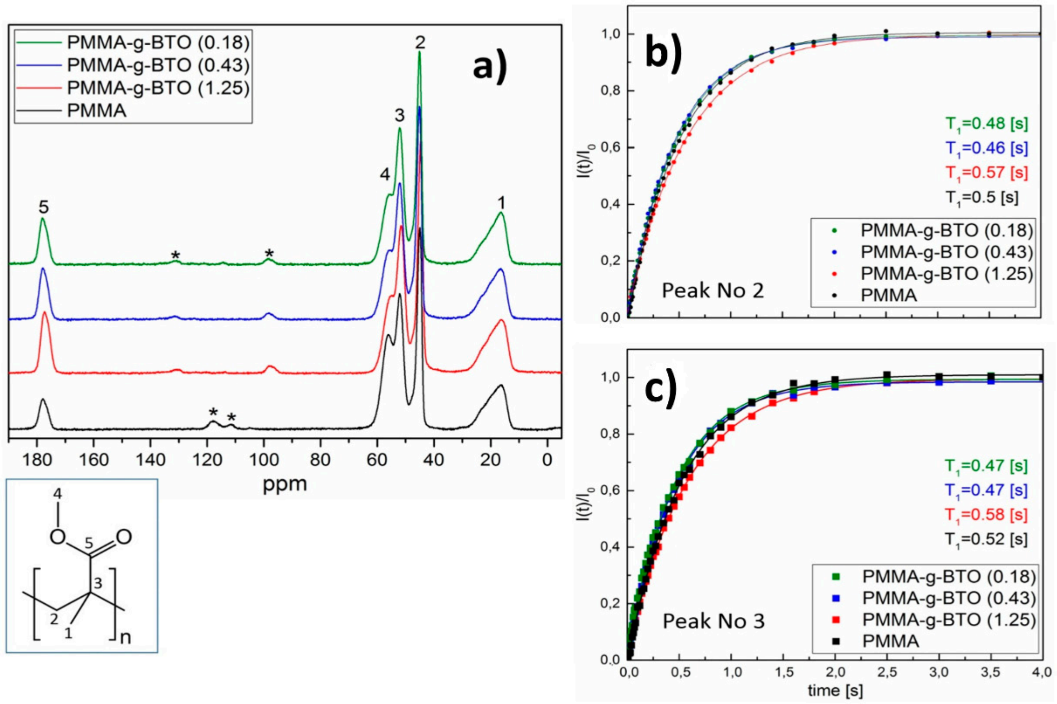


**Figure S8.** Spin-lattice relaxation time $T_1$ measurements. $^{13}C$ CP/MAS NMR spectra recorded at 20 °C for the investigated systems (**a**); Magnetization recovery for protons measured using the cross-polarization method at 20 °C for two individual signals attributed to methylene carbons 2 (**b**), and quaternary carbons 3 (**c**).

**Table S1.** Sample specification of core-shell BTO-g-PMMA composites. Calculated values of ε for composites, according to Lichtenecker rule, were obtained taking into account BTO density equal 6.02 g/cm$^3$ and its ε = 180.

| Sample name (grafting density) | % f.inorg (weight fraction) | % f.inorg (volume fraction) | Measured ε of composite | Calculated ε of composite (Lichtenecker) |
|---|---|---|---|---|
| BTO-*g*-PMMA (1.25) | 33 | 9 | 4.4 | 4.3 |
| BTO-*g*-PMMA (0.43) | 54 | 19 | 5.9 | 6.4 |
| BTO-*g*-PMMA (0.18) | 62 | 24 | 8.0 | 8.1 |
